# Favorable outcome of a gastrocutaneous fistula managed with EndoVAC therapy post-bariatric surgery: a case report

**DOI:** 10.1093/jscr/rjaf839

**Published:** 2025-10-21

**Authors:** Martin Islas Torres, Jose Orozco Álvarez Malo, José Maria Zepeda Torres, Luis Osvaldo Suárez Carreón, Francisco Javier Plascencia Posada, Rodrigo Prieto Aldape, José Víctor Pérez Navarro, José Abraham Flores Cardoza, Rodrigo Hernández Ramírez, Andrea Michelle Salas Carlock

**Affiliations:** General Surgery, Centro Medico Nacional de Occidente IMSS, Hospital de Especialidades, Av. Belisario Domínguez 1000, Belisario Domínguez, Guadalajara, Jalisco 44329, Mexico; Bariatric Surgery, Instituto Nefrologico de Tijuana, Misión de San Ignacio 4428, Zona Urbana Rio Tijuana, Tijuana, Baja California 22010, Mexico; General Surgery, Centro Medico Nacional de Occidente IMSS, Hospital de Especialidades, Av. Belisario Domínguez 1000, Belisario Domínguez, Guadalajara, Jalisco 44329, Mexico; General Surgery, Centro Medico Nacional de Occidente IMSS, Hospital de Especialidades, Av. Belisario Domínguez 1000, Belisario Domínguez, Guadalajara, Jalisco 44329, Mexico; General Surgery, Centro Medico Nacional de Occidente IMSS, Hospital de Especialidades, Av. Belisario Domínguez 1000, Belisario Domínguez, Guadalajara, Jalisco 44329, Mexico; Bariatric Surgery, Hospital Jardines, Av Manuel J. Clouthier 669, Jardines de Guadalupe, Zapopan, Jalisco 45030, Mexico; General Surgery, Centro Medico Nacional de Occidente IMSS, Hospital de Especialidades, Av. Belisario Domínguez 1000, Belisario Domínguez, Guadalajara, Jalisco 44329, Mexico; General Surgery, Centro Medico Nacional de Occidente IMSS, Hospital de Especialidades, Av. Belisario Domínguez 1000, Belisario Domínguez, Guadalajara, Jalisco 44329, Mexico; General Surgery, Centro Medico Nacional de Occidente IMSS, Hospital de Especialidades, Av. Belisario Domínguez 1000, Belisario Domínguez, Guadalajara, Jalisco 44329, Mexico; General Surgery, Centro Medico Nacional de Occidente IMSS, Hospital de Especialidades, Av. Belisario Domínguez 1000, Belisario Domínguez, Guadalajara, Jalisco 44329, Mexico

**Keywords:** endoluminal vacuum therapy, gastrocutaneous fistula, bariatric surgery, minimally invasive treatment, wound healing

## Abstract

This case report details the successful management of a gastrocutaneous fistula following bariatric surgery using endoluminal vacuum-assisted closure therapy (EVT/EVAC). Recognized increasingly in recent literature as an effective minimally invasive approach, EVT promotes fistula closure through continuous negative pressure applied via endoscopically placed sponges, facilitating wound drainage, tissue granulation, and defect healing. The patient’s favorable outcome underscores EVT’s potential as a safe alternative to surgical repair, especially in complex or refractory cases. A review of current evidence highlights high success rates and safety profiles for EVT in managing postbariatric leaks and fistulas, emphasizing its growing role in endoscopic therapeutic strategies.

## Introduction

Management of a gastrocutaneous fistula following bariatric surgery with endoluminal vacuum-assisted closure therapy (EVT), also known as endoscopic vacuum therapy (EVAC), is gaining increasing support in the medical literature as a safe and effective modality, especially for leaks and fistulas refractory to conventional treatments. This innovative approach involves the endoscopic placement of an open-pore polyurethane sponge or similar device, connected to a nasogastric or drainage tube, directly within the fistula tract or cavity, promoting continuous wound drainage, tissue granulation, and fistula closure [1–5]. In this report, we describe an interesting and challenging case managed successfully with EVT, and we provide a comprehensive review of current evidence supporting its use in similar clinical scenarios, emphasizing its potential as a minimally invasive alternative to surgery.

## Case report

**Figure 1:**
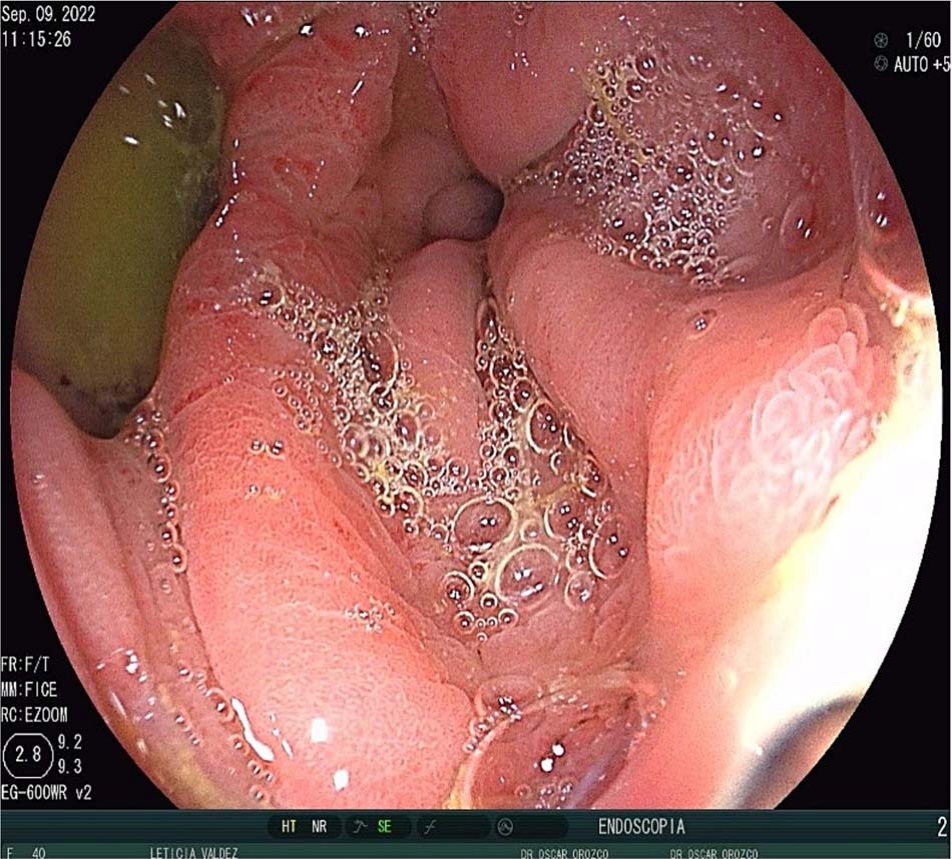
Gastrocutaneous fistula.

**Figure 2:**
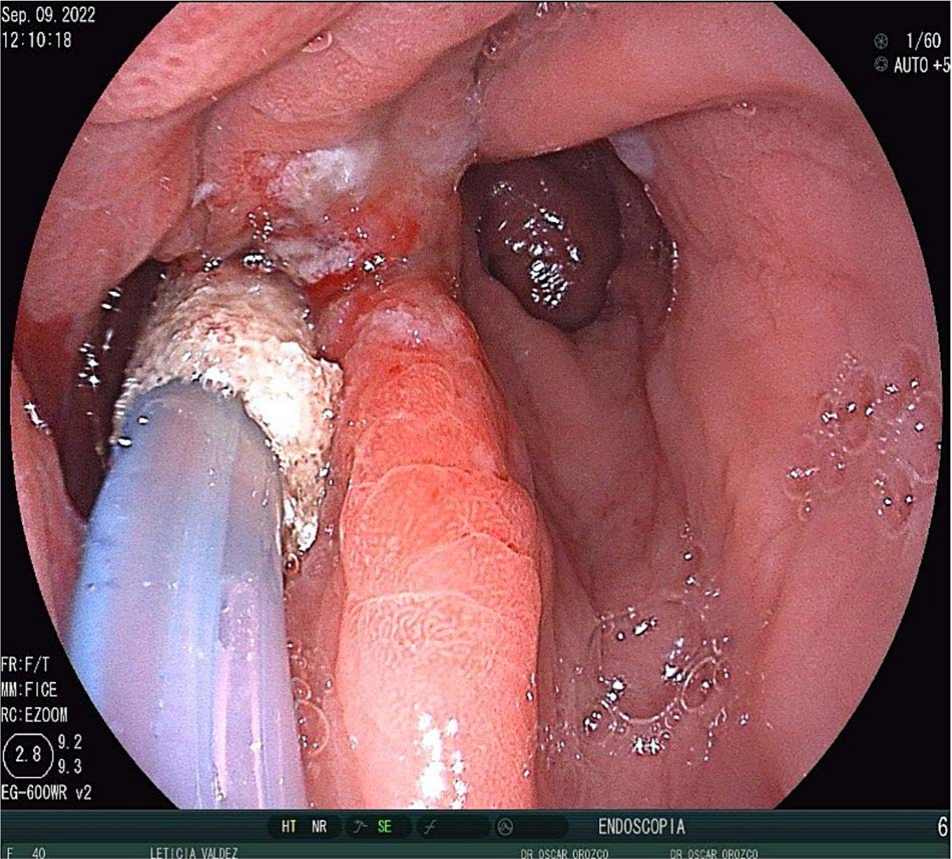
EndoVAC therapy.

**Figure 3:**
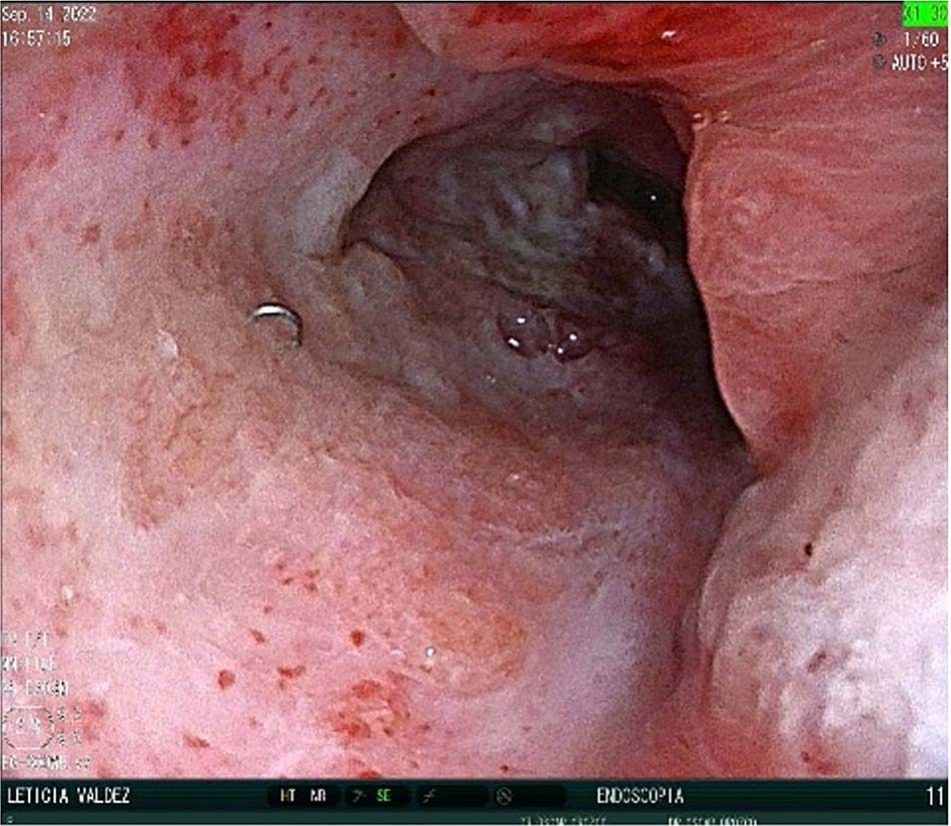
Post-EndoVAC therapy. Closure of the fistula is seen.

**Figure 4:**
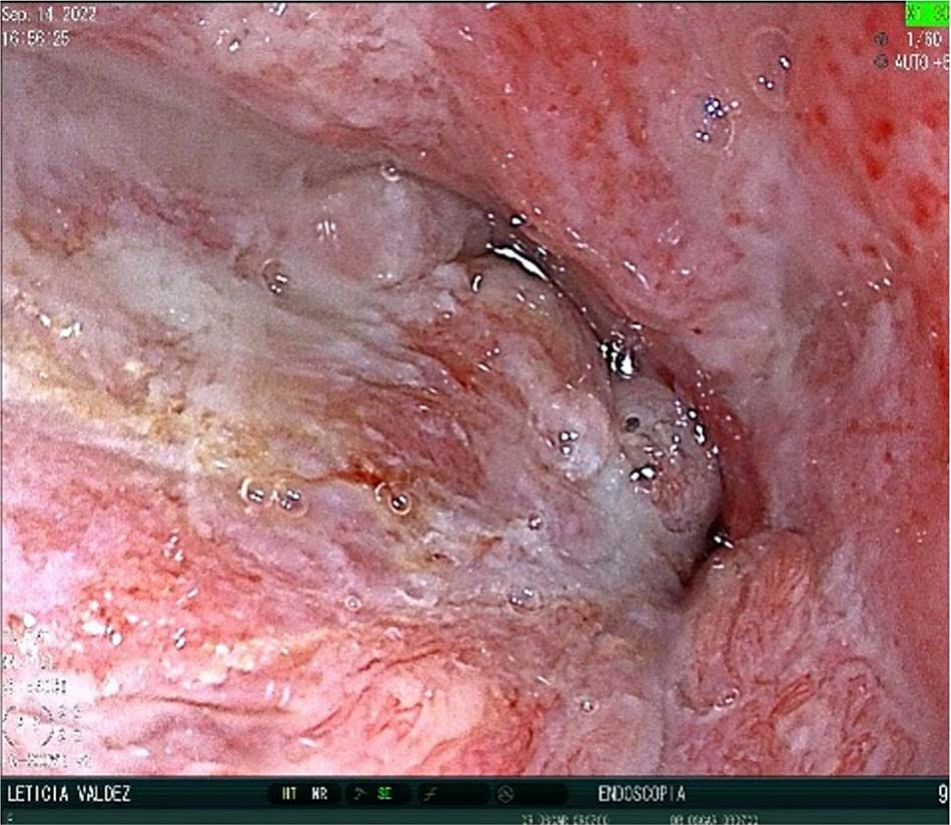
Close-up post-EndoVAC therapy. Closure of the fistula is seen.

A 40-year-old female patient was readmitted on postoperative day (POD) 28 following a gastric sleeve procedure, presenting with a low-output gastrocutaneous fistula. Her medical history included type 2 diabetes mellitus and hypertension. Her postoperative course was complicated by an intra-abdominal collection, leading to the placement of a percutaneous pigtail drain on POD 75, followed by a diagnostic laparoscopy for adhesiolysis and drain removal on POD 112. Upon the current admission, she remained hemodynamically stable, tolerating a liquid diet and parenteral nutrition, with no signs of peritoneal irritation or acute abdomen. Physical examination revealed a conscious, oriented patient with a soft, depressible, and nontender abdomen; a visible fistula with purulent, foul-smelling, yellow-green secretion; and no signs of systemic infection, with a mild fever of 37.4°C. Ultrasonography identified a simple, anechoic collection measuring 42 × 24 × 33 mm (volume ~18 cc) located in the left subcostal region, with no other intra-abdominal fluid collections; additionally a figure consistent with the fistulous tract (Fig. 1) and the presence of the pig tail catheter were observed, with prior contrast studies indicating postsurgical changes without evidence of leaks. The decision was made to manage the fistula conservatively with EndoVAC (endoscopic vacuum-assisted closure) therapy, which involved the placement of a specialized sponge (Fig. 2) connected to a vacuum device through the fistula tract to promote continuous wound drainage, reduce the bacterial load, and facilitate tissue granulation and closure of the fistula. The system was initiated on POD 30, and over a total therapy duration of 28 days, four sponge exchanges were performed at 3-day intervals. This approach successfully promoted granulation and drainage, leading to complete fistula closure confirmed on POD 58 (Figs 3 and 4). The patient was discharged in stable condition, with outpatient follow-up planned for continued monitoring.

## Discussion

The EVT technique involves endoscopic placement of an open-pore polyurethane sponge or a similar device, connected to a nasogastric or drainage tube, directly into the fistula tract or cavity. The system is then connected to continuous negative pressure (typically −75 to −125 mmHg), which promotes drainage, reduces local contamination, collapses the fistula cavity, and stimulates granulation tissue formation, thereby facilitating closure of the fistula tract [1–5]. The device is exchanged endoscopically every 3–5 days, with the number of exchanges and total duration of therapy depending on the size and chronicity of the fistula and the patient’s clinical response [1, 6]. Median dwell times in published series range from ~17 to 26 days, with a mean of 6–7 sponge exchanges [1, 3, 4].

Clinical success rates for EVT in the management of postbariatric leaks and fistulas are high, with systematic reviews and cohort studies reporting closure rates of 87%–100% [1–4]. EVT can be used as a primary therapy or as salvage therapy after failure of surgical or stent-based interventions [2, 3]. Early initiation of EVT is associated with shorter treatment duration and hospital stay [3]. EVT is particularly advantageous in cases where local peritonitis or compartmentalized collections are present, as it provides both internal drainage and direct wound therapy [2, 4].

Adverse events are relatively uncommon, with reported rates of moderate complications of ~6% and system dislodgement in 12%–13% of cases [1]. EVT does not preclude subsequent surgical or endoscopic interventions if required [4]. In select cases of chronic or complex fistulae, adjunctive techniques such as combined endoscopic–percutaneous vacuum therapy and plug insertion have been described, though these require further validation [7].

## Conclusion

This case exemplifies the evolving role of endoluminal vacuum-assisted closure therapy as a minimally invasive and effective treatment for gastrocutaneous fistulas post-bariatric surgery, especially in cases refractory to conventional management. The successful resolution in this patient underscores EVT’s high efficacy, safety, and versatility in promoting fistula closure through continuous wound drainage, tissue granulation, and rapid recovery, while minimizing the need for invasive surgical intervention. The current literature supports its use in selected patients—particularly those with chronic, complex, or difficult-to-manage fistulas—offering a promising alternative with high success rates and low complication profiles. As the demand for less invasive approaches grows, further studies and standardized protocols are essential to optimize patient selection, treatment duration, and combination strategies, ensuring EVT remains a cornerstone in the management of postbariatric fistulas.
